# Sexual Harassment Among Medical residents and interns in Sfax: Impact and correlated factors

**DOI:** 10.1192/j.eurpsy.2025.2290

**Published:** 2025-08-26

**Authors:** S. Ajmi, F. Cherif, O. Bouattour, D. Mnif, I. Feki, R. Masmoudi, J. Masmoudi

**Affiliations:** 1 Psychiatry A department, Hedi Chaker hospital university, Sfax, Tunisia

## Abstract

**Introduction:**

Despite global movements to combat Sexual harassment (SH), it continues to affect individuals in various professions, with medical residents and interns being particularly vulnerable.

**Objectives:**

To explore the prevalence of SH among medical trainees in Sfax, Tunisia.

To evaluate the consequences of SH on this group and factors related to SH.

**Methods:**

We conducted a cross-sectional and descriptive study involving medical residents and interns working in hospitals in Sfax. Data were collected using an anonymous self-questionnaire. This questionnaire was published on social media during January and February 2024. It included sociodemographic characteristics, psychiatric and medical history, psychoactive substance use, professional data, information related to sexual life and experiences related SH.

**Results:**

We collected 141 responses, of which 19.9% declined to participate in this study.

Finally, a total of 113 participants, with an average age of 27.92 years, were recruited. The sex ratio (M/F) was equal to 0.54. In our population, 20.4% were interns. Among the participants, 68.1% were single, 91.2% were from urban backgrounds and 12.4% had psychiatric follow-up. Among the participants, 41.6% reported experiencing sexual harassment during their practice at the hospitals in Sfax. The most common form self-reported as harassment was verbal harassment (43.3%).

In our study, most victims of sexual harassment (SH) tried to ignore (36.2%) and avoid (34%) the harasser. Some participants noted that they were afraid of career repercussions.

Regarding the consequences of SH, sleep disorders and the feeling of burnout were the most frequently reported medical effects, with a prevalence of 10.6% in both cases. Additionally, the onset or increase in tobacco consumption (8%) was the most commonly reported addictive consequence.

On a sexual level, 16 participants (14.2%) reported experiencing negative impacts on their sexuality. Sexual desire disorders (8.8%) were the most frequently reported sexual consequences, followed by a decrease in sexual satisfaction (2.7%).

In our survey, SH was statistically more prevalent among female participants (p <0.001) and among participants with a low or middle socioeconomic status (p = 0.036).

**Conclusions:**

These results highlight the urgent need for hospitals to implement preventive measures, support victims, and promote a safer working environment for medical trainees.

**Disclosure of Interest:**

None Declared

